# Prevalence, awareness, treatment, and control of hypertension in older people: results from the population-based KORA-age 1 study

**DOI:** 10.1186/s12889-020-09165-8

**Published:** 2020-07-02

**Authors:** Samuel Muli, Christa Meisinger, Margit Heier, Barbara Thorand, Annette Peters, Ute Amann

**Affiliations:** 1 Institute for Medical Informatics, Biometry, and Epidemiology, Ludwig-Maximilians-Universität München, Munich, Germany; 2 Helmholtz Zentrum München, German Research Center for Environmental Health (GmbH), Independent Research Group Clinical Epidemiology, Ingolstädter Landstraße 1, 85764 Neuherberg, Germany; 3 Chair of Epidemiology, Ludwig-Maximilians-Universität München, UNIKA-T, Augsburg, Germany; 4 Helmholtz Zentrum München, German Research Center for Environmental Health (GmbH), Institute of Epidemiology, Neuherberg, Germany; 5 University Hospital of Augsburg, KORA Study Centre, Augsburg, Germany

**Keywords:** Prevalence, Awareness, Treatment, Control, Hypertension, Older adults

## Abstract

**Background:**

Hypertension remains a significant modifiable risk factor for cardiovascular diseases and a major determinant of morbidity and mortality. We aimed to describe sex-stratified age-standardized estimates of prevalence, awareness, treatment and control of hypertension, and their associated factors in older adults.

**Methods:**

The KORA-Age1 is a population-based cross-sectional survey carried out in 2008/2009 on individuals aged 65–94 years in Augsburg region, Germany. Blood pressure measurements were available for 1052 out of 1079 persons who participated in the physical examination. Factors associated with prevalence, awareness and control of hypertension were investigated by multivariable logistic regression.

**Results:**

The overall prevalence of hypertension (≥140/90 mmHg) was 73.8% [95% confidence interval (CI), 69.3–77.9], representing 74.8% (95% CI, 68.4–80.2) in men and 73.5% (95% CI, 66.8–79.3) in women. Among those with hypertension, 80.2% (95% CI, 75.3–84.4) were aware of their hypertensive condition and 74.4% (95% CI, 69.2–79.1) were on treatment for hypertension. Among those aware of their hypertension status, 92.8% (95% CI, 88.8–95.6) were on treatment and 53.7% (95% CI, 47.0–60.1) had their blood pressure controlled. Hypertension was more frequent in individuals who were older, obese, or had diabetes. Higher education attainment or presence of comorbidities was associated with higher level of hypertension awareness. Individuals taking three antihypertensive drug classes were more likely to have controlled hypertension compared with those taking one antihypertensive drug class, odds ratio (OR), 1.85 (95% CI, 1.14–2.99).

**Conclusion:**

Our findings identified high prevalence of hypertension and relevant health gaps on awareness, treatment and suboptimal control of hypertension in older adults in Germany. Screening for hypertension should especially target older adults with low educational attainment and ‘healthy’ elderly with less contact to physicians.

## Background

Cardiovascular diseases (CVDs) are a leading cause of premature death and morbidity, and a major global public health concern [1]. In 2016, it was estimated that 17.9 million people died from CVDs, which represented 31% of all-cause mortality in that year [2]. Hypertension is widely recognized as the most significant modifiable risk factor for CVDs [3], and a leading contributor of disability-adjusted life years [4]. Moreover, hypertension remains a significant concern for public health systems due to the high prevalence of its associated risk factors. Physical inactivity, alcohol consumption, smoking, and poor dietary habits are highly prevalent in the general population, yet have a lifelong bearing on the risk of hypertension [5].

The incidence of hypertension significantly rises with increasing age [6, 7]; hence, it is more prevalent among older adults than in young and middle-aged population [4]. Numerous population-based surveys in various regions of the world estimate that 7 out of 10 adults, 65 years and older, have been diagnosed with elevated blood pressure [8–10]. This trend is likely to persist, as the population of older persons in high-income countries continues to grow [11]. In some countries such as the U.S., even though the trends in incidence of hypertension have only slightly changed [12], the absolute number of people with hypertension is ever growing due to the aging population [13]. Some studies observed that about 24–30% of older adults with hypertension were not aware of their hypertension status [9, 14, 15], and about 32% were not on treatment [9, 15]. In the same studies, the proportion of treated and controlled hypertension was barely 50% [9, 14, 15], which suggests that treatment and controlled hypertension in older adults is suboptimal — and an issue of great public health interest. In Germany, epidemiological studies on prevalence, awareness, treatment and control of hypertension have only been conducted in adults aged 18–79 years [16], but so far not in the very old population. Therefore, the aim of this study is to determine the prevalence, awareness, treatment and control of hypertension, and their associated factors in older people aged 65–94 years from the general population in Germany.

## Methods

### Study design and population

The KORA-Age1 study was carried out in 2008/2009 within the framework of the multidisciplinary Cooperative Health Research in the Augsburg Region (KORA) on individuals aged 65–94 [17]. Briefly, 5991 persons of the previous four KORA surveys conducted between 1985 and 2001, who were born in 1943 or earlier, still alive and living in the study region, were mailed a brief self-administered questionnaire between November 2008 and September 2009. Furthermore, an extended telephone interview was conducted on 4127 study participants to collect more specific data on morbidity and health status (e.g., kidney and heart diseases). A sex- and age-stratified subsample (100 persons per stratum) of 2005 persons were invited for physical examination (e.g., blood pressure measurements); 1079 (53.8%) participated, and of these, 963 persons were examined at the KORA study center, 94 were examined during a home visit, and 22 got only a short interview [15]. Detailed information on study design and sampling is provided elsewhere [18, 19].

### Study variables

#### Measurement of blood pressure and anti-hypertensive medications

Three blood pressure measurements were performed on the right arm (after a rest of at least 5 min), within an interval of 3 min, in a seated position using an automatic digital oscillometer (HEM 705CP-II Omron Corporation Japan). Cuffs were adjusted appropriately for arm girth of the participant. The mean of the second and third measurements was determined and adopted as the blood pressure reading for the present study. All blood pressure measurements were performed on a single occasion.

All participants were requested to provide medications used within the last 7 days preceding the examination appointment. Medications in KORA studies were assigned as ‘antihypertensive medications’ only if the compounds taken were classified by the most recent guidelines of the German Hypertension Society [20]. In addition, categorization by antihypertensive drug classes based on the German anatomic therapeutic chemical (ATC) classification was used for the following: diuretics (C03, C02L, C07B-C07D, C08G, C09BA or C09DA), beta blockers (C07), angiotensin converting enzyme (ACE) inhibitors (C09A or C09B), angiotensin receptor blockers (ARBs) (C09C or C09D), renin-inhibitors (C09XA), calcium channel blockers (CCBs) (C08, C07FB22, C09BB or C09DB) and other antihypertensives (CO2 without C02KX, C02KP (phytotherapy) and C02KH (homeopathy or anthroposophy)).

#### Definitions

The study adopted widely recognized standard definitions for the study outcomes, namely hypertension, awareness, treatment, and control of hypertension.

#### Hypertension

Participants were identified as hypertensive if they had systolic blood pressure (SBP) ≥ 140 mmHg or diastolic blood pressure (DBP) ≥90 mmHg or their blood pressure was controlled as a result of taking antihypertensive medications. Since these medications may also be indicated for health conditions other than hypertension, information on antihypertensive medication use was only utilized for identifying hypertensive cases if the person confirmed to be aware of hypertension. Similar approach is applied in other studies in Germany [5, 21].

#### Awareness

Individuals with hypertension who responded with “yes” to the following question: “have you ever been diagnosed by a doctor or health professional with elevated blood pressure?” were considered as being aware of their hypertension.

#### Treatment

Hypertensives aware of their hypertension diagnosis, who were on at least one of the antihypertensive medications as identified above.

#### Control

Treated hypertensives who at the time of measurements had their blood pressure below the target goals i.e. SBP < 140 mmHg and DBP < 90 mmHg.

#### Special patient groups

Stronger blood pressure target goals for high-risk patient groups such as diabetes are recommended in some guidelines [22]. In a subgroup analysis considering this group, controlled hypertension was defined as blood pressure below or equal to 130/80 mmHg [22].

### Covariates

Besides age and sex, the following covariates were considered and described in detail. Educational attainment levels were classified according to highest academic qualification, namely low level (‘Hauptschule’), middle level (‘Realschule’), high level (‘Abitur’ or higher level). Smoking status was categorized into current smoker (for individuals who smoke regularly or irregularly), former smokers, and never smokers. Height and weight of participants were measured during physical examination, and the body mass index (BMI) calculated by dividing weight by height squared (kg/m^2^), and classified according to the World Health Organization (WHO) guidelines: underweight: < 18.5, normal ≥18.5 and < 25, overweight ≥25 and < 30, and obese ≥30. Additionally, physical activity was assessed using standardized interviews in which participants reported their weekly leisure-time physical activity during summer and winter. Those who reported almost no sports activity or about 1 h per week irregularly, were grouped as inactive. Participants who reported leisure-time physical activity of 1 h or more per week regularly in winter and summer, were grouped as active. Comorbidity status of participants was self-reported for specific conditions such as heart and kidney diseases, diabetes mellitus, previous stroke, and cancer diagnosis in the last 3 years. For diabetes mellitus, use of antidiabetic medications (A10) additionally contributed to the final allocation as diabetic person. If participant was unable to answer interview questions, a proxy interview was conducted.

### Statistical analyses

Descriptive statistical analyses were performed to characterize the distribution of blood pressure, antihypertensive medication and drug classes, and to determine the prevalence, awareness, treatment and control of hypertension. Estimates of these study outcomes (per 100 persons) were age-standardized using the German population as on 31.12.2009. 10-year age groups were created and population weights for each group determined. Since a specific population weight for individuals aged above 90 years was not available, the nearest population weight (85 years and above) was used. To explore group differences between individuals with hypertension and those without, student t-tests and Mann–Whitney U tests were applied appropriately on continuous variables, and chi-squared test on categorical variables. Association of covariates and the prevalence, awareness, treatment and control of hypertension were explored using logistic regression analyses. In developing the logistic regression models, four dependent variables were identified: hypertension status among all participants (yes = 1, no = 0), awareness among hypertensive individuals (aware = 1, unaware = 0), treatment among aware hypertensives (treated =1, untreated =0), and control of blood pressure among treated hypertensives (controlled = 1, uncontrolled = 0). This analysis approach is reported in other studies [7, 14, 15, 23]. Since there was no significant interaction between sex and the four dependent variables (p < 0.05), the logistic regression models were not stratified by sex. Purposeful selection of covariates for building multivariable logistic regression models was conducted, beginning with univariate analysis, each covariate at a time [24]. Covariates were included in multivariable models if univariate analysis showed level of significance of p-value < 0.25 [24] or if they are documented in literature as potential confounders. Analyses of variance and likelihood ratio tests were also performed on the adjusted multivariable models. The “treatment among aware” model (treated = 601, untreated =37) was dropped due to the small sample size of the untreated group in terms of events per variable (EPV), which showed poor predictive performance. The acceptability of the multivariable models was evaluated using the Pearson-Chi-Square goodness of fit test. P-values < 0.05 were considered statistically significant. The R program 3.2.3 was used to conduct the analyses.

## Results

Out of the 1079 physically examined study participants, blood pressure measurements were available for 1052 persons, representing 50.3% men and 49.7% women. Out of these, 790 participants were identified as hypertensive (397 males and 393 females), representing unweighted prevalence of 75.1%. Table 1 summarizes the demographic and clinical characteristics of the population stratified by hypertension status. The participants’ age ranged between 65 and 94 years, with mean age of 75.9 years (SD = 6.6). There was a significant age difference between hypertensives and non-hypertensives, representing means of 76.2 (SD = 6.4) and 75.1 (6.9) years respectively (p = 0.020). Additionally, there were significant differences in the BMI for participants with hypertension who had a median BMI of 28.3 (IQR = 25.8–31.2) compared to 26.9 (IQR = 24.8–29.5) in those without hypertension (p < 0.001). There was a significantly higher frequency of obesity and diabetes in individuals with hypertension compared to those without hypertension, representing a 34.1% vs. 20.6%, *p* < 0.001 and 21.2% vs. 7.6%, *p* < 0.001, respectively.

**Table 1:** Characteristics of the study participants stratified by hypertension status

Variable	Total (*n* = 1052)	Hypertension (*n* = 790)	No Hypertension (*n* = 262)	*P*-value^†^
Sex				1.000
Male	529 (50.3)	397 (50.3)	132 (50.4)	
Female	523 (49.7)	393 (49.7)	130 (49.6)	
Age (mean (±SD))	75.9 (6.6)	76.2 (6.4)	75.1 (6.9)	0.020
Age (years) (%)				0.020
65–69	227 (21.6)	157 (19.9)	70 (26.7)	
70–74	227 (21.6)	163 (20.6)	64 (24.4)	
75–79	241 (22.9)	192 (24.3)	49 (18.7)	
80–84	232 (22.1)	186 (23.5)	46 (17.6)	
85–94	125 (11.9)	92 (11.6)	33 (12.6)	
Living with partner (%)	656 (63.0)	420 (79.7)	236 (45.9)	< 0.001
Smoking status (%)				< 0.001
Never	565 (53.7)	417 (52.8)	148 (56.5)	0.504
Ex-smoker	438 (41.6)	337 (42.7)	101 (38.5)	
Current smoker	49 (4.7)	36 (4.6)	13 (5.0)	
Education ^a^ (%)				0.112
Low level	717 (68.2)	552 (69.9)	165 (63.0)	
Middle level	190 (18.1)	136 (17.2)	54 (20.6)	
High level	145 (13.8)	102 (12.9)	43 (16.4)	
BMI (kg/m^2^) (%)				< 0.001
Underweight (< 18.5)	1 (0.1)	0 (0.0)	1 (0.4)	
Normal (18.5–24.9)	213 (20.2)	142 (18.0)	71 (27.1)	
Overweight (25–29.9)	515 (49.0)	379 (48.0)	136 (51.9)	
Obese (≥30)	323 (30.7)	269 (34.1)	54 (20.6)	
BMI (median (IQR))	27.9 (25.4–30.8)	28.3 (25.8–31.2)	26.9 (24.8–29.5)	< 0.001
Physically active (%) ^b^	561 (53.3)	422 (53.4)	139 (53.1)	0.975
Heart diseases (%) *	327 (31.1)	250 (31.6)	77 (29.4)	0.544
Kidney diseases (%) *	50 (4.8)	42 (5.3)	8 (3.1)	0.185
Diabetes (%) ^c^	187 (17.8)	167 (21.2)	20 (7.6)	< 0.001
Stroke (%) *	88 (8.4)	68 (8.6)	20 (7.6)	0.715

†*p* value obtained from comparing differences between hypertensives and non-hypertensives using X^2^-test for categorical variables, t-test for age difference, and Mann-Whitney U test for BMI medians

^a^ Based on the highest academic qualification: Low level (‘Hauptschule’), Middle level (‘Realschule’), High level (‘Abitur’ or higher level)

^b^ Physical activity: self-reported sporting activity during leisure time of 1 h or more per week, regularly or irregularly

^c^ Diabetes mellitus was both self-reported and confirmed also through use of antidiabetic medications

* Self-reported comorbidities

*SD* Standard deviation of the observed age in our sample; *BMI* Body mass index; *IQR* Interquartile range

### Prevalence, awareness, treatment and control of hypertension

**Fig. 1:**
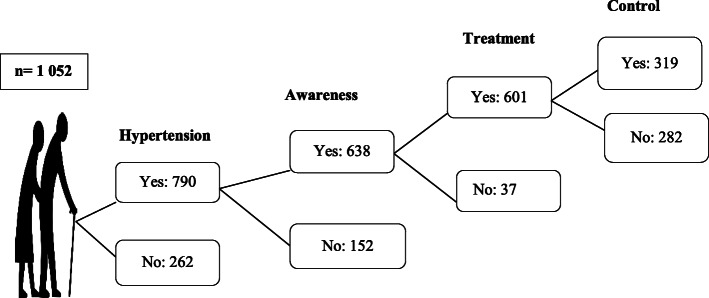
Flow diagram showing the frequency, awareness, treatment and control of hypertension in KORA-Age 1 study

Figure 1 provides a schematic representation of the frequency, awareness, treatment, and control of hypertension in the KORA Age-1 study.

As summarized in Table 2, the overall age-standardized prevalence of hypertension was 73.8% (95% CI: 69.3–77.9), with men having slightly higher prevalence of hypertension than women (74.8% vs. 73.5%). The frequency of hypertension was lowest in the group 65–74 years with 70.5% (95% CI: 66.0–74.6) and highest in participants aged 75–84 years with 79.9% (95% CI: 76.0–83.4). Among individuals with hypertension (n = 790), 80.2% (95% CI: 75.3–84.4) affirmed having been diagnosed with hypertension; hence, were considered as being aware of their hypertension status. The level of awareness increased gradually with age, and was higher in women than in men (81.3% vs. 78.3%).

**Table 2:** Prevalence, awareness, treatment and control of hypertension by age and sex

	Age (years)	Prevalence (*n* = 1052)	Awareness (*n* = 790)	Treatment among aware hypertensives (*n* = 638)	Control among treated (*n* = 601)
Male	65–94	74.8 (68.4–80.2) ^a^	78.3 (71.1–84.2) ^a^	93.9 (87.9–97.1) ^a^	52.5 (43.2–61.5) ^a^
Female	65–94	73.5 (66.8–79.3) ^a^	81.3 (74.0–87.0) ^a^	92.6 (86.1–96.4) ^a^	54.9 (45.3–64.2) ^a^
Total	65–74	70.5 (66.0–74.6)	78.4 (73.5–82.7)	90.8 (86.4–93.9)	54.4 (47.7–60.9)
	75–84	79.9 (76.0–83.4)	82.0 (77.7–85.7)	96.1 (93.2–97.8)	53.0 (47.2–58.8)
	85–94	73.6 (64.8–80.9)	83.7 (74.2–90.3)	97.4 (90.1–99.5)	49.3 (37.7–61.0)
	65–94	73.8 (69.3–77.9) ^a^	80.2 (75.3–84.4) ^a^	92.8 (88.8–95.6) ^a^	53.7 (47.0–60.2) ^a^

^a^Age-standardized according to the distribution of the Germany population, 31 December 2009

Overall, in individuals with hypertension, 74.4% (95% CI: 69.2–79.1) were on antihypertensive treatment, and the frequency of treated hypertension increased with age. However, considering treatment only among those aware of their status, 92.8% (95% CI, 88.8–95.6) were on antihypertensive medication, and 53.7% (95% CI: 47.0–60.1) of the treated hypertensives had attained blood pressure below 140/90 mmHg. The frequency of optimal blood pressure control slightly declined with age, and was lowest in the oldest group, 49.3% (95% CI: 37.7–61.0).

**Fig. 2:**
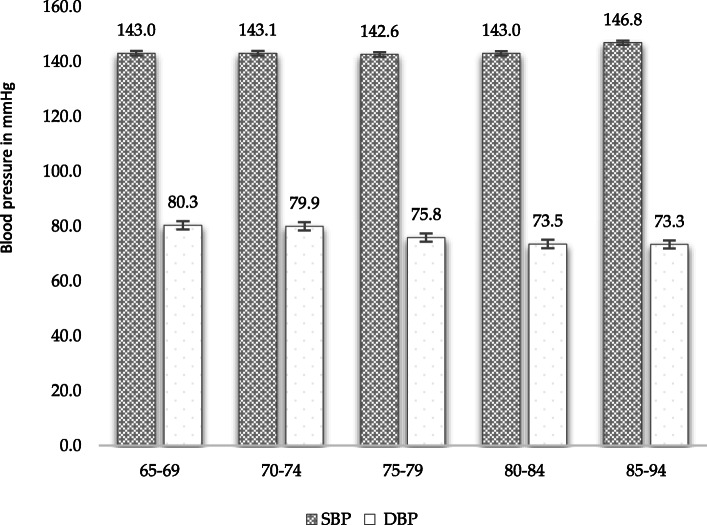
Distribution of systolic and diastolic blood pressure by 5-year age groups among hypertensives (*n* = 790). Abbreviations: DBP – Diastolic blood pressure, SBP – systolic blood pressure

The distribution of SBP and DBP by 5-year age groups is summarized in Fig. 2. The oldest group, 85–94 years, had the highest SBP mean of 146.8 mmHg, but had the lowest DBP mean of 73.3 mmHg.

### Results of multivariable analysis of factors associated with prevalence, awareness and control of hypertension

The results of the multivariable logistic models are summarized in Table 3. Briefly, the prevalence of hypertension increased with increasing age for age group 75–79 years (OR 1.79, 95% CI: 1.11–2.72), and for age group 80–84 years (OR 1.95, 95% CI: 1.22–3.12), and declined beyond the age of 85 years, (OR 1.41, 95% CI: 0.80–2.47), *p* = 0.2337 in comparison to age group 65–69 years. There was higher frequency of hypertension in individuals with obesity compared with those with normal weight (OR 2.14, 95% CI: 1.39–3.29). Individuals with diabetes were also more likely to have hypertension compared to non-diabetic persons (OR 2.82, 95% CI: 1.70–4.70). There were no statistically significant differences in hypertension prevalence with respect to other sociodemographic characteristics such as sex, living with partner, education attainment, and lifestyle factors such as smoking status and physical activity.

**Table 3:** Results of the multivariable logistic regression models on factors associated with prevalence, awareness and control of hypertension

Characteristics/variables	Prevalence (*n* = 1039)	Awareness (*n* = 780)	Control (*n* = 595)	Control^c^ (*n* = 595)
	OR^a^ (95%, CI)	OR^a^ (95%, CI)	OR^a^ (95%, CI)	OR^a^ (95%, CI)
Sex (ref: Men)	1	1	1	1
Women	1.11 (0.79–1.56)	1.21 (0.78–1.90)	1.00 (0.66–1.49)	1.03 (0.36–1.96)
Age Groups (years) (ref: ≥65–69)	1	1	1	1
70–74	1.09 (0.72–1.67)	1.12 (0.64–1.96)	1.20 (0.70–2.06)	0.97 (0.56–1.68)
75–79	1.79 (1.11–2.72) *	1.12 (0.64–1.95)	1.18 (0.69–2.02)	0.97 (0.57–1.66)
80–84	1.95 (1.22–3.12) **	1.19 (0.66–2.15)	0.91 (0.53–1.56)	0.70 (0.40–1.21)
85–94	1.41 (0.80–2.47)	1.26 (0.59–2.69)	0.81 (0.42–1.58)	0.78 (0.40–1.54)
Living with Partner (ref: No)	1	1	1	1
Yes	1.01 (0.72–1.41)	0.94 (0.61–1.46)	0.93 (0.63–1.37)	0.83 (0.56–1.54)
Education (ref: Low)	1	1	1	1
Middle level	0.88 (0.60–1.29)	1.58 (0.93–2.66)	1.55 (0.98–2.44)	1.59 (1.01–2.51) *
High level	0.78 (0.51–1.18)	2.40 (1.20–4.80) *	0.84 (0.51–1.37)	0.91 (0.55–1.51)
Smoking Status (ref: Never)	1	1	1	1
Ex-smoker	1.19 (0.86–1.65)	0.76 (0.50–1.16)	1.19 (0.82–1.74)	1.12 (0.76–1.64)
Current smoker	1.21 (0.59–2.49)	0.58 (0.24–1.38)	2.31 (0.90–5.92)	2.36 (0.93–5.99)
BMI (ref: Normal)	1	1	1	1
Underweight (< 18.5) ^b^	0.00 (0.00 - ∞)	–	–	–
Overweight (25–30)	1.33 (0.93–1.91)	1.44 (0.88–2.36)	0.81 (0.50–1.34)	0.87 (0.53–1.43)
Obese (≥30)	2.14 (1.39–3.29) ***	1.97 (1.14–3.42) *	1.02 (0.61–1.73)	1.09 (0.64–1.85)
Physical Activity (ref: No)	1	1	1	1
Yes	1.26 (0.92–1.72)	0.85 (0.57–1.27)	0.89 (0.63–1.26)	1.04 (0.73–1.48)
Heart Disease (ref: No)	1	1	1	1
Yes	0.94 (0.68–1.31)	2.38 (1.46–3.87) ***	1.19 (0.83–1.72)	1.35 (0.93–1.97)
Stroke (ref: No)	1	1	1	1
Yes	1.01 (0.58–1.75)	4.45 (1.36–14.62) *	0.99 (0.57–1.73)	0.95 (0.54–1.67)
Kidney disease (ref: No)	1	1	1	1
Yes	1.94 (0.88–4.30)	1.85 (0.62–5.48)	1.31 (0.64–2.71)	1.02 (0.49–2.12)
Diabetes (ref: No)	1	1	1	1
Yes	2.82 (1.70–4.70) ***	2.05 (1.17–3.58) *	0.86 (0.57–1.29)	0.38 (0.25–0.58) ***
Drug combinations^c^ (ref: [1])	–	–	1	1
2 classes	–	–	1.35 (0.85–2.12)	1.32 (0.83–2.09)
3 classes	–	–	1.85 (1.14–2.99) *	1.90 (1.17–3.10) **
≥ 4 classes	–	–	1.24 (0.68–2.27)	1.22 (0.66–2.27)

Abbreviations: *CI* Confidence interval; *OR* Odds ratio; *BMI* Body mass index expressed as kg/m^2^;

*p value < 0.05; **p values < 0.01; ***p value < 0.001

^a^The odds ratio from multivariable logistic regression, adjusted for other variables in the table

^b^ only one individual was underweight and was observed to be non-hypertensive. The OR for this category is therefore, unreliable

^**c**^ Subgroup analysis in which controlled hypertension was defined as blood pressure ≤ 130/80 mmHg for individuals with diabetes mellitus

The level of education had significant influence on awareness status. Individuals with the highest educational attainment were more likely to be aware of their blood pressure status compared to individuals with low education (OR 2.40, 95% CI: 1.20–4.80). Having comorbidities was also associated with higher odds of being aware: obesity (OR 1.97, 95% CI: 1.14–3.42), heart disease (OR 2.38, 95% CI: 1.46–3.87), stroke (OR 4.45, 95% CI: 1.36–14.62), and diabetes (OR 2.05, 95% CI: 1.17–3.58), compared to individuals without the respective comorbid status.

There were no statistically significant differences with respect to sociodemographic characteristics or presence of comorbidities and control of blood pressure. However, treated hypertensives were more likely to have controlled blood pressure if under three antihypertensive drug classes (OR 1.85, 95% CI: 1.14–2.98) compared to those on monotherapy. Taking more than three antihypertensive drug classes was not associated with statistically significant higher odds of being controlled (OR 1.24, 95% CI: 0.68–2.27), *p* = 0.490 compared to monotherapy.

### Diabetes and blood pressure control

Multivariable adjusted regression analysis considering controlled hypertension among diabetic individuals as blood pressure ≤ 130/80 mmHg, showed that treated hypertensives with diabetes were less likely to have controlled blood pressure (OR 0.38, 95% CI: 0.25–0.58) compared to non-diabetic hypertensives. As shown in Table 3, there was no statistically significant change in the rest of covariates of the hypertension control model.

## Discussion

This study showed that the prevalence of hypertension in adults aged 65–94 years was 73.8%, translating to 3 out of 4 older adults having high blood pressure. The age-standardized prevalence of hypertension was slightly higher in men than women, and increased with age. Even though our data showed highest SBP in individuals aged 85–94 years, a guarded approach in clinical interpretation of these findings should be considered. Unlike in the middle-aged persons, high SBP in the very elderly (> 85 years) is not consistently associated with increased risk for CVD events [24, 25].

Being aware of one’s hypertension status may be a motivation for positive lifestyle modification: increasing physical activity, moderating alcohol consumption, smoking cessation, and dietary changes. Since 80.2% of hypertensive persons in this study were aware of their hypertension status, and 92.8% of those aware were on treatment, this suggests that the burden of untreated hypertension might be attributed to lack of awareness. Considering evidence that treating hypertension in older persons markedly reduces the risk of cardiovascular complications and mortality [25], intensifying screening of hypertension in the elderly should therefore, be encouraged.

The present study showed that the proportion of controlled hypertension in the older German population is quite low – 53.7%. We however, hypothesized that measuring blood pressure on a single occasion potentially underestimated the proportion of controlled hypertension. A 24-h ambulatory blood pressure monitoring, though hardly ever used in population-based observational studies, may detect fluctuations in blood pressure and other symptoms in treated hypertensive subjects [13]. Our observations could also indicate a high prevalence of resistant hypertension in the population. In part, this may be due to non-adherence to treatment, which is common in older individuals [13]. Moreover, drug induced elevation of blood pressure was not evaluated in our study. It should be noted that some medications such as nonsteroidal anti-inflammatory drugs (NSAIDs) significantly elevate blood pressure [26] and have been shown to destabilize the effect of main classes of antihypertensive drugs [27].

### Factors associated with prevalence, awareness, and control of hypertension

The odds ratios for the prevalence of hypertension as shown in Table 3, suggests that the frequency of hypertension gradually increased with increasing age up to the age of 84 years, then declined with further ages. Other studies in comparable settings have reported similar observations [15, 23, 28]. Individuals who reach a very old age above 85 years are generally healthier, and this may explain the declining prevalence of hypertension in the oldest age group.

Obese individuals were about twice more likely to have hypertension compared to those with healthy weight. Numerous studies have reported higher frequency of hypertension in overweight and obese subjects [9, 15, 23, 28]. However, even though obesity may be an independent predictor of hypertension, there is potential interaction with other lifestyle factors such as physical activity and dietary habits.

Expectedly, individuals with high education attainment had more than twice higher odds of being aware of their hypertension status than those with low education. Education is a well elucidated determinant of health disparity, and such disparities have been shown to be more pronounced in later life phases [29]. Our findings underscore the need to target individuals with low educational attainment for hypertension screening and treatment.

There was also a higher level of awareness in individuals with other comorbidities such as obesity, heart diseases, stroke, and diabetes. These findings are consistent with other studies [15, 23, 28]. We considered that individuals with these comorbidities are more likely to have higher frequency visits to their treating physicians and are preferentially screened for hypertension.

The poor control of hypertension as observed in our study reflects difficulties in blood pressure control in later life phases, and similar findings have been reported in other studies [9, 14, 23, 28]. Hypertensive participants who were taking multiple drug classes were more likely to attain blood pressure control targets compared to individuals on a single antihypertensive drug class. Beyond three combinations, further clinical effect was not statistically significant. It could be interpreted that individuals who were taking more than three antihypertensive drug classes probably had advanced hypertension or resistant hypertension that was more difficult to be controlled by treatment. These findings are supported by a recent clinical trial, that reported better control of blood pressure in individuals treated with more than one antihypertensive drug class as initiation therapy compared to those treated with single drug class or stepped-care approach [30]. The European Society of Hypertension (ESH) 2018 guidelines recently recognized that, monotherapy treatment approach was generally ineffective for high risk hypertensive patient groups – those in grade 2 and 3 hypertension – and recommends multiple antihypertensive drug treatment, starting with two antihypertensive drug classes and up-titrating to three antihypertensive drug classes when hypertension control goals are not attained [31]. However, it should be emphasized that treatment goals for hypertension in the elderly poses significant challenges due to age-related physiological alterations, frailty and presence of multiple comorbidities [11, 32], and adverse health events associated with polypharmacy [33]. As such, individualized treatment strategy for the elderly is encouraged due to their special health conditions.

When blood pressure cut-off points for patients with diabetes were considered – as demonstrated in the subgroup analysis model – individuals with diabetes were shown to have significant difficulties with blood pressure control compared to non-diabetic individuals. Poor control of blood pressure among diabetic individuals is documented in other epidemiological studies [15, 23]. Several complex and inter-related biological changes may explain this potential resistance to antihypertensive drugs. Diabetes, which is believed to exacerbate stiffening of arteries, is also associated with higher risk of renal impairment and cardiovascular complications [34]. These complications have negative implications on blood pressure control. Moreover, volume overload which is commonly reported in persons with diabetes and associated with diabetic nephropathy, is believed to cause resistant hypertension [35]. However, other authors posit that difficulties in control of blood pressure in persons with diabetes maybe a result of inadequate clinical management, particularly, inadequate and inappropriate antihypertensive medication doses, drug class combinations, and poor compliance [36].

### Implications for practice and research

There is need to improve screening of elderly population groups, especially those with a low level of education and those perceived to be at high risk of hypertension due to obesity or diabetes. Early treatment of hypertension may prevent irreversible arterial damage especially, in older persons who are at higher risk for vascular injury [5], and reduce risk of stroke and heart attack [25]. It should be noted, however, that treatment of hypertension especially in the very old, should be considered on the balance between risk and benefits for individual patients [11]. Evidence on the ideal blood pressure targets and safest hypertension regimen for this age group remains inconclusive [11, 25, 37]. Nonetheless, the importance of adequate clinical management of hypertension including multiple antihypertensive drug treatment especially in older adults with diabetes should be considered.

### Strengths and limitations

The study methods were highly standardized and consistent with widely recognized guidelines. As a population-based study, we recruited noninstitutionalized participants and employed comprehensive medication use review procedures. Our findings are therefore, plausible and comparable with similar studies. However, the blood pressure measurements were performed on a single visit; hence, white-coat hypertension and masked hypertension could be possible [38]. As a result, there might be a potential overestimation of prevalence of hypertension and underestimation of the controlled hypertension. Moreover, consistent with the general limitations of cross-sectional studies, the possibility of residual confounding as well as potential overestimation of some associations due to use of odds ratios instead of prevalence ratios [39] cannot be ruled out. Some of our variables were self-reported and could not be objectively verified at the time of the study.

## Conclusion

Our study identified relevant health gaps on awareness, treatment and control of hypertension in older people aged 65–94 years in Germany. Screening for hypertension would particularly be useful among older adults with low educational attainment, who were observed to have low awareness of their hypertension status. Even though we observed that individuals taking three classes of antihypertensive drugs were more likely to have controlled hypertension compared to those taking a single class of antihypertensive drugs, a guarded, more individualized treatment approach should be considered when dealing with elderly subjects.

## Data Availability

The full dataset supporting the conclusions of this article is available upon request and application from the Cooperative Health Research in the Region Augsburg (KORA; https://www.helmholtz-muenchen.de/kora/ueber-kora/index.html).

## Abbreviations

BMI: Body mass index
CVD: Cardiovascular diseases
CI: Confidence Interval
DBP: Diastolic blood pressure
EPV: Events per variable
IQR: Interquartile range
KORA: Cooperative health research in the Augsburg Region
OR: Odds ratio
SBP: Systolic blood pressure
SD: Standard deviation
